# Symmetric collateral pattern on CTA predicts favorable outcomes after endovascular thrombectomy for large vessel occlusion stroke

**DOI:** 10.1371/journal.pone.0284260

**Published:** 2023-05-04

**Authors:** Robert W. Regenhardt, Michael H. Lev, Julian He, Adam A. Dmytriw, Justin E. Vranic, James D. Rabinov, Christopher J. Stapleton, Aman B. Patel, Aneesh B. Singhal, R. Gilberto Gonzalez

**Affiliations:** 1 Department of Neurosurgery, Massachusetts General Hospital, Harvard Medical School, Boston, MA, United States of America; 2 Department of Neurology, Massachusetts General Hospital, Harvard Medical School, Boston, MA, United States of America; 3 Department of Radiology, Massachusetts General Hospital, Harvard Medical School, Boston, MA, United States of America; 4 Athinoula A Martinos Center for Biomedical Imaging, Boston, MA, United States of America; 5 Mass General Brigham Data Science Office, Boston, MA, United States of America; Foshan Sanshui District People’s Hospital, CHINA

## Abstract

Endovascular thrombectomy (EVT) has revolutionized large vessel occlusion (LVO) stroke management, but often requires advanced imaging. The collateral pattern on CT angiograms may be an alternative because a symmetric collateral pattern correlates with a slowly growing, small ischemic core. We tested the hypothesis that such patients will have favorable outcomes after EVT. Consecutive patients (n = 74) with anterior LVOs who underwent EVT were retrospectively analyzed. Inclusion criteria were available CTA and 90-day modified Rankin Scale (mRS). CTA collateral patterns were symmetric in 36%, malignant in 24%, or other in 39%. Median NIHSS was 11 for symmetric, 18 for malignant, and 19 for other (p = 0.02). Ninety-day mRS ≤2, indicating independent living, was achieved in 67% of symmetric, 17% of malignant, and 38% of other patterns (p = 0.003). A symmetric collateral pattern was a significant determinant of 90-day mRS ≤2 (aOR = 6.62, 95%CI = 2.24,19.53; p = 0.001) in a multivariable model that included age, NIHSS, baseline mRS, thrombolysis, LVO location, and successful reperfusion. We conclude that a symmetric collateral pattern predicts favorable outcomes after EVT for LVO stroke. Because the pattern also marks slow ischemic core growth, patients with symmetric collaterals may be suitable for transfer for thrombectomy. A malignant collateral pattern is associated with poor clinical outcomes.

## Introduction

There is an urgent need to expand endovascular thrombectomy (EVT) treatment for patients with large vessel occlusion (LVO) ischemic stroke [1]. While LVO stroke accounts for the largest proportion of stroke-related death and disability [2], the acute care of these patients has been revolutionized by EVT [3–5]. One approach to increase treatment is to identify patients who may benefit despite delays accompanying transfer to an EVT-capable center [6–8]. These ‘slow progressor’ patients may constitute a third or more of transferred patients. While they can be identified with advanced imaging such as diffusion MRI and CT perfusion (CTP) [9–11], these resources are not often available at community hospitals and underserved regions [12–14]. This is especially true for patients in the extended window or with unknown onset [15]. We recently demonstrated that patients who are slow progressors are identified by a symmetric collateral pattern on CTA [16].

Collaterals are alternative vessels, consisting of primary circle of Willis and secondary pial-pial leptomeningeal anastomoses, that can compensate for reduced blood flow in the setting of LVO [2]. Collateral patterns vary dramatically among patients with stroke and are highly related to infarct growth [17]. Indeed, the collateral pattern assessed by presentation CT angiography (CTA) may be an appropriate proxy for infarct volume and infarct growth rate [18]. Among patients with LVO not treated with reperfusion therapies, symmetric collateral pattern on CTA had a sensitivity of 87% and a specificity of 94% for 24-hour infarct volume <50 cc [16]. Herein, we tested the hypothesis that LVO patients will have better clinical outcomes after EVT if they have symmetric collaterals.

## Methods

This study was approved by the Massachusetts General Hospital institutional review board. All research was performed in accordance with relevant guidelines and regulations. Informed consent was waived based on minimal patient risk and practical inability to perform the study without the waiver. Consecutive patients who underwent EVT for anterior circulation LVO over two years were identified from a prospectively maintained database at a single referral center [19]. Inclusion criteria were anterior circulation LVO involving the internal carotid (ICA), middle cerebral (MCA) M1 segment, or MCA M2 segment, treatment with EVT, available CTA for review, and prospectively recorded 90-day modified Rankin Scale (mRS) score. There were no missing data for included patients. Exclusion criteria were lack of LVO, posterior circulation occlusion, anterior cerebral (ACA) occlusion, MCA M3 segment occlusion or distal, and not selected for treatment with EVT. Local treatment selection guidelines during the study period stated that patients who met the following criteria were “likely to benefit”: last known well <24 hours, NIHSS ≥6, ASPECTS ≥6 within 6 hours, and significant mismatch by CTP/MRI within 6–24 hours. Patients who met the following were considered “unlikely to benefit”: last known well >24 hours, NIHSS <4, baseline mRS >3, limited life expectancy, ASPECTS ≤4 within 6 hours, and no mismatch or core >120mL by CTP/MRI [20].

CTA was performed using multi-detector scanners (GE Medical Systems) from the vertex to the aortic arch following injection of 65–140 ml of a nonionic contrast agent (Isovue; Bracco Diagnostics) at a rate of 3 to 4 ml/s. The median parameters were 1.25-mm slice thickness, 220 mm reconstruction diameter, 120 kV, and 657 mA. CTDI_vol_ ranged from 65–95 mGY, and DLP ranged from 2593–3784 mGY-cm.

**Fig 1:**
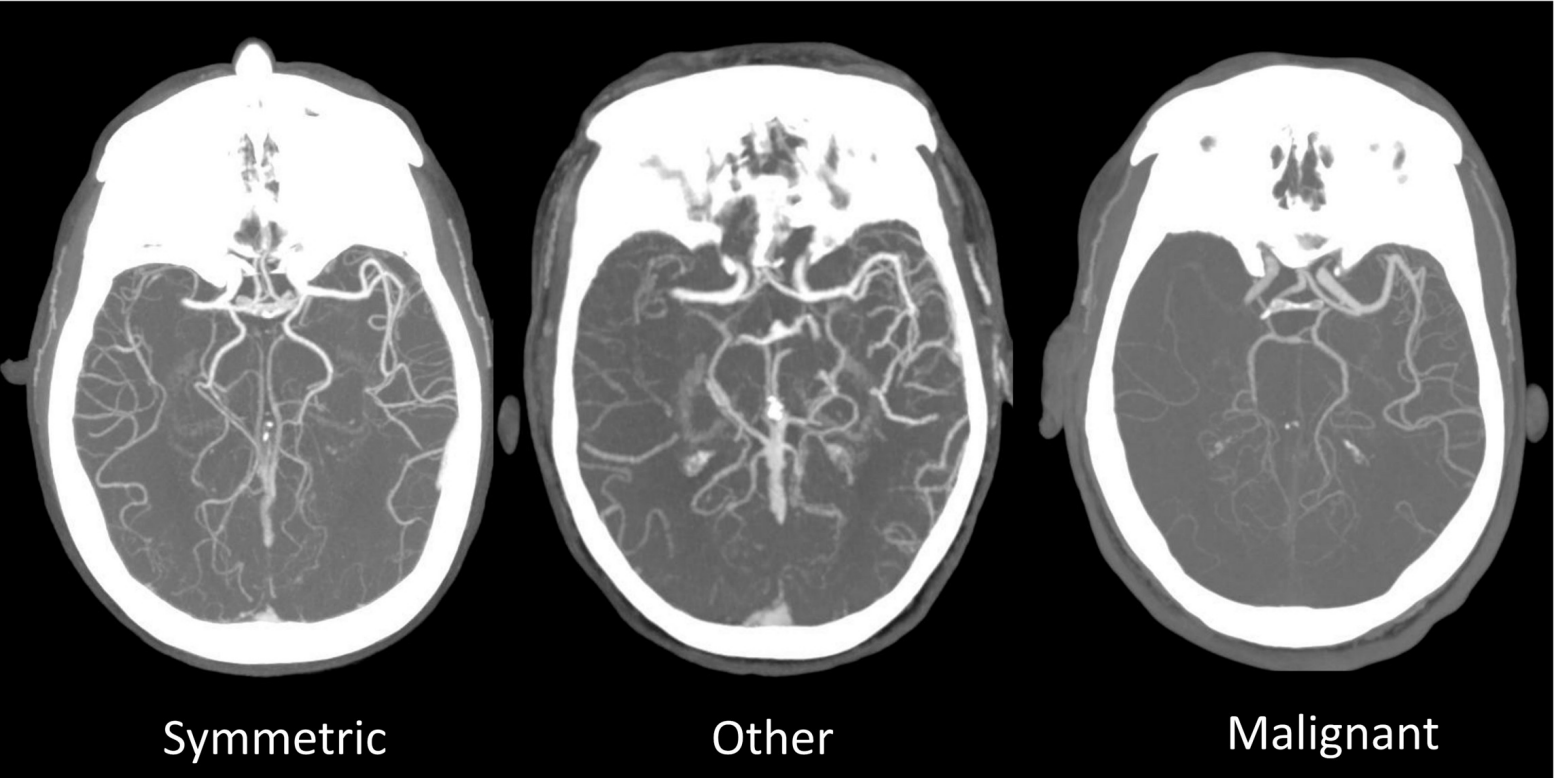
Collateral patterns assessment and classification system. A symmetric pattern was defined as contrast visualized with similar conspicuity of the ischemic compared to the contralateral non-ischemic MCA territory. A malignant pattern was defined as no contrast visualized over at least 50% of the MCA territory at risk. “Other” was defined as any additional pattern, rated as intermediate between symmetric and malignant.

Collateral patterns were interpreted by CAQ-certified neuroradiologists with over 25 years’ experience interpreting acute stroke studies (RGG, MHL). Interpreters were blinded to clinical presentations, treatments, other imaging, and clinical outcomes. Patterns were determined by visual review of the maximum intensity projection arterial phase CTA images, which were classified as “symmetric”, “malignant”, or “other” [16]. Briefly, a symmetric pattern was defined as contrast visualized with similar or near similar conspicuity of the ischemic compared to the contralateral non-ischemic MCA territory. A malignant pattern was defined as no contrast visualized over at least 50% of the MCA territory at risk. “Other” was defined as any additional pattern, rated as intermediate between symmetric and malignant (Fig 1). The vessel occlusion site on CTA was documented as internal carotid artery (ICA) terminus, first (M1) middle cerebral artery (MCA) segment, and second (M2) MCA segment [21]. Cervical ICA stenosis was defined as >70% by NASCET criteria [22].

The local database included demographic information, medical history, clinical presentation, treatments, and outcomes for consecutive patients treated with EVT. Stroke severity (National Institutes of Health Stroke Scale, NIHSS) was determined as described [23]. Alteplase treatment decisions were guideline-based at the discretion of a vascular neurologist [24]. EVT treatment decisions were guideline-based at the discretion of the treating vascular neurologist and neurointerventionalist. Thrombolysis in cerebral infarction (TICI) scores were determined by a neurointerventionalist using the modified scale: 2a partial filling <50%, 2b partial filling ≥50%, 3 complete perfusion [4]. Successful reperfusion was defined as TICI 2b-3 [3]. Symptomatic intracerebral hemorrhage (sICH) was defined as any PH1 or PH2 by ECASS criteria associated with new symptoms during the hospitalization [25]. 90-day modified Rankin Scale (mRS) was obtained by telephone call or follow-up clinic visit [26, 27]. Good functional outcome was defined as 90-day mRS ≤2 [28].

Median and interquartile range (IQR) were reported for continuous nonparametric variables. Percent and count were reported for categorical variables. Differences among three groups of nonparametric continuous variables were assessed using the Kruskal Wallis test. Associations with good functional outcome were assessed by logistic regression. Variables of interest were selected *a priori* for their possible relevance to good functional outcome. Distributions were assumed nonparametric based on the Kolmogorov-Smirnov and Shapiro-Wilk tests. Two-tailed P values <0.05 were considered statistically significant. Analyses were performed with Prism version 6.01 (GraphPad) and SPSS version 23.0 (IBM Corp).

## Results

Among 74 patients who met inclusion criteria from 2019 to 2020, the median age was 75 (IQR 58–82), and 49% were female (Table 1). Examples of the collateral grading system are shown in Fig 1.

**Table 1:** Patient demographics, risk factors, presentations, treatments, and outcomes.

	Malignant (n = 18)	Other (n = 29)	Symmetric (n = 27)	Total	P-Value
Age, Median (IQR)	71 (52,82)	76 (59,81)	72 (59,81)	75 (58,82)	0.72
Female	7 (39%)	16 (55%)	13 (48%)	36 (49%)	0.55
Black	2 (11%)	1 (3%)	1 (4%)	4 (5%)	0.47
White	11 (61%)	16 (55%)	22 (82%)	49 (66%)	0.10
Asian	1 (6%)	3 (10%)	1 (4%)	5 (7%)	0.60
Hispanic	1 (6%)	1 (3%)	1 (4%)	3 (4%)	0.93
Baseline mRS ≥3	2 (11%)	2 (7%)	1 (4%)	5 (7%)	0.62
Atrial Fibrillation	5 (28%)	8 (28%)	8 (30%)	21 (28%)	0.98
Coronary Disease	7 (39%)	11 (38%)	5 (19%)	23 (31%)	0.21
Diabetes Mellitus	3 (17%)	9 (31%)	7 (26%)	19 (26%)	0.55
Dyslipidemia	6 (33%)	11 (38%)	13 (48%)	30 (41%)	0.57
Heart Failure	4 (22%)	7 (24%)	3 (11%)	14 (19%)	0.42
Hypertension	13 (72%)	18 (62%)	21 (78%)	52 (70%)	0.43
Obesity/Overweight	6 (33%)	14 (48%)	11 (41%)	31 (42%)	0.59
Previous Stroke	3 (17%)	2 (7%)	6 (22%)	11 (15%)	0.27
Previous TIA	1 (6%)	1 (3%)	2 (7%)	4 (5%)	0.81
Chronic Renal Insufficiency	2 (11%)	3 (10%)	4 (15%)	9 (12%)	0.87
Smoker	1 (6%)	6 (21%)	2 (7%)	9 (12%)	0.19
Cardioembolism	9 (50%)	18 (62%)	15 (56%)	42 (57%)	0.71
Large Artery Atherosclerosis	3 (17%)	2 (7%)	5 (19%)	10 (14%)	0.40
Cryptogenic	3 (17%)	4 (14%)	7 (26%)	14 (19%)	0.49
Dissection	2 (11%)	0 (0%)	0 (0%)	2 (3%)	0.04
Hypercoagulability	1 (6%)	5 (17%)	0 (0%)	6 (8%)	0.06
NIHSS, Median (IQR)	18 (14,23)	19 (12,22)	11 (8,18)	17 (10,22)	0.02
Intravenous Thrombolysis	6 (33%)	11 (38%)	4 (15%)	21 (28%)	0.14
Telestroke Consult	6 (33%)	12 (41%)	17 (63%)	35 (47%)	0.11
Transfer	8 (44%)	15 (52%)	20 (74%)	43 (58%)	0.10
LKW-Telestroke	1.38 (0.99,1.73)	1.53 (1.10,3.25)	3.27 (1.23,6.00)	1.73 (1.10,4.17)	0.18
LKW-Hub Arrival	2.59 (1.81,3.25)	2.63 (0.67,4.72)	4.47 (2.24,6.68)	3.08 (0.90,5.07)	0.14
LKW-Arterial Puncture	3.42 (2.82,4.22)	3.03 (2.22,5.37)	5.25 (2.72,7.79)	3.60 (2.70,6.07)	0.16
Intracranial ICA Occlusion	5 (28%)	1 (3%)	3 (11%)	9 (12%)	0.04
M1 MCA Occlusion	13 (72%)	23 (79%)	15 (56%)	51 (69%)	0.15
M2 MCA Occlusion	0 (0%)	5 (17%)	9 (33%)	14 (19%)	0.02
Stent Retriever EVT	8 (44%)	10 (39%)	8 (31%)	26 (37%)	0.64
Aspiration Only EVT	10 (56%)	16 (62%)	18 (69%)	44 (63%)	0.64
TICI 2b-3	15 (83%)	26 (90%)	24 (89%)	65 (88%)	0.80
sICH	3 (17%)	1 (3%)	1 (4%)	5 (7%)	0.16
Discharge mRS ≤2	1 (6%)	3 (10%)	9 (33%)	13 (18%)	0.02
90-Day mRS ≤2	3 (17%)	11 (38%)	18 (67%)	32 (43%)	0.003

Abbreviations: IQR Interquartile Range, mRS modified Rankin Scale, TIA Transient Ischemic Attack, NIHSS National Institutes of Health Stroke Scale, LKW Last Known Well, ICA Internal Carotid Artery, MCA Middle Cerebral Artery, EVT Endovascular Thrombectomy, TICI Thrombolysis in Cerebral Infarction, sICH symptomatic Intracerebral Hemorrhage.

Amongst our EVT-treated patients, collaterals were symmetric in 36%, malignant in 24%, or other in 39%. Comparing patients with these collateral patterns, there were no differences in demographics, risk factors, time from last known well, thrombolysis treatment, successful TICI 2b-3 reperfusion, or symptomatic intracranial hemorrhage. Median NIHSS was 11 (IQR 8–18) for symmetric, 18 (IQR 14–23) for malignant, and 19 (IQR 12–22) for other (p = 0.02). Intracranial ICA occlusions were present in 11% of symmetric, 28% of malignant, and 3% of other patterns (p = 0.04) (Table 1).

**Fig 2:**
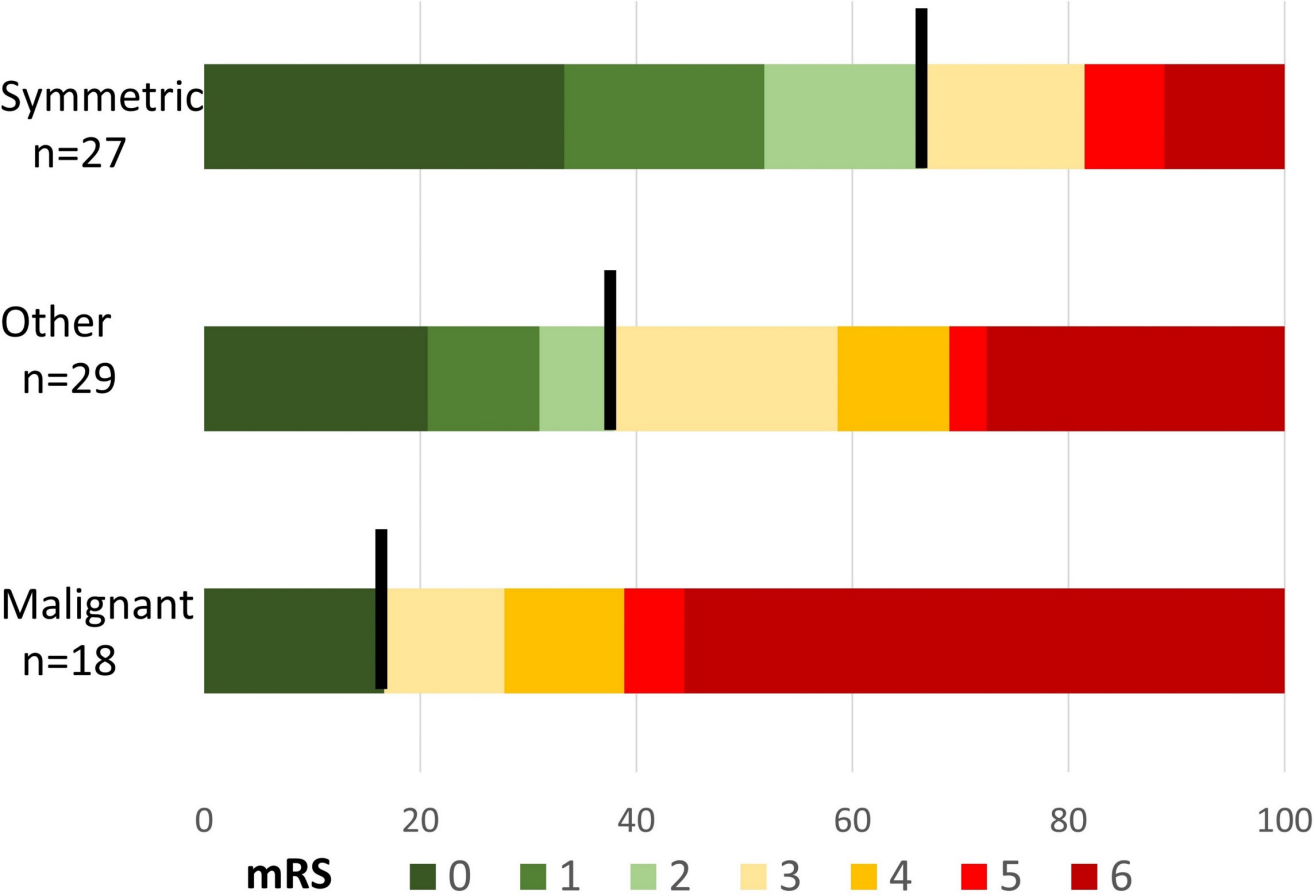
Ninety-day modified Rankin Scale (mRS) score comparing collateral patterns.

Ninety days after thrombectomy, patients were living independently (mRS ≤2) in 67% of patients with symmetric collaterals. Only 17% of those with malignant collaterals had favorable outcomes after 3 months while over 50% were deceased. Only 38% of those with the other pattern had 90-day mRS ≤2. These differences are highly significant (p = 0.003) (Table 1 and Fig 2).

The patient collateral pattern was also a significant determinant of 90-day mRS ≤2 (aOR = 6.62, 95%CI = 2.24,19.53; p = 0.001) in a multivariable model that included age (aOR = 0.92, 95%CI = 0.87,0.97; p = 0.001), NIHSS (aOR = 0.98, 95%CI = 0.89,1.08; p = 0.68), baseline mRS ≥3 (aOR = 6.14, 95%CI = 0.60,63.12; p = 0.13), intravenous thrombolysis (aOR = 2.14, 95%CI = 0.52,8.91; p = 0.29), occlusion location (aOR = 0.53, 95%CI = 0.16,1.82; p = 0.31), and successful TICI 2b-3 reperfusion (aOR = 10.45, 95%CI = 1.05,104.3; p = 0.05) (Table 2).

**Table 2:** Determinants of 90-day modified Rankin Scale (mRS) score ≤2.

	Univariable OR (95% CI)	P-Value	Multivariable aOR (95% CI)	P-Value
Collaterals	3.19 (1.57,6.47)	0.001	6.62 (2.24,19.53)	0.001
Age	0.96 (0.93,0.99)	0.007	0.92 (0.87,0.97)	0.001
NIHSS	0.97 (0.90,1.04)	0.36	0.98 (0.89,1.08)	0.68
Baseline mRS ≥3	0.87 (0.14,5.52)	0.88	6.14 (0.60,63.12)	0.13
Intravenous Thrombolysis	0.74 (0.27,2.09)	0.58	2.14 (0.52,8.91)	0.29
Occlusion Location	0.97 (0.42,2.23)	0.95	0.53 (0.16,1.82)	0.31
TICI 2b-3	3.00 (0.58,15.55)	0.19	10.45 (1.05,104.3)	0.05

Abbreviations: NIHSS National Institutes of Health Stroke Scale, TICI Thrombolysis in Cerebral Infarction, OR Odds Ratio, CI Confidence Interval.

## Discussion

We found that LVO patients with symmetric collaterals have better outcomes after EVT. We have previously shown that most LVO patients with this pattern are slow progressors; they have a small ischemic core at presentation that remains small for at least 24 hours. Together with our previous work [9, 16], we demonstrate that symmetric collaterals identify LVO patients that are slow progressors likely to benefit from EVT despite time delays associated with patient transfer. Currently, there are no accepted guidelines for transferring patients for EVT. Various approaches have been used within different stroke networks, ranging from transferring all patients with LVO to using advanced imaging (MRI, CTP) for patient selection. Here we propose an alternative that uses widely available CTA.

In the present analysis of patients who underwent EVT, there was significant variability in collateral patterns. They were symmetric in 36%, malignant in 24%, and other in 39%. This is consistent with our local EVT treatment selection guidelines, which do not exclude patients based on collateral patterns. Indeed, others have reported a range of collateral quality, albeit assessed differently, among patients treated with EVT [29, 30]. In addition, there was a trend for patients with malignant patterns to have shorter times from last known well (LKW), but this difference did not reach statistical significance. This may be related to our treatment exclusion of patients with poor ASPECTS or large infarcts since patients with poor collaterals have faster infarct growth [16]. Furthermore, patients with more proximal occlusion locations were more likely to have poor collaterals; ICA occlusions were present in 28% of malignant, 3% of other, and 11% of symmetric patients. This stands to reason, and early studies have explored the relationship between occlusion location and collateral quality [31].

We show that symmetric collaterals were associated with less severe stroke presentations. Among our cohort, the median NIHSS was 18 for malignant patterns, 19 for other, and 11 for symmetric. Other studies corroborate our results, showing that patients with poor collaterals have higher NIHSS [18, 32]. This also stands to reason as the ischemic hemispheres have less perfusion and are more likely to cause clinical symptoms [2].

Importantly, a symmetric collateral pattern is highly associated with good 90-day functional outcomes after EVT. Malignant collaterals, however, were most frequently associated with poor clinical outcomes. Ninety-day mRS ≤2 (living independently) was achieved in 67% of patients with symmetric, 17% with malignant, and 38% with other collateral patterns. This relationship persisted even when controlling for age, stroke severity, baseline disability, thrombolysis treatment, occlusion location, and successful reperfusion after EVT. Indeed, there has been prior investigation into other predictors of outcome after thrombectomy. One model highlighted that reperfusion status, 24-hour NIHSS, and sICH were predictors of early mortality after EVT [33]. Another analysis demonstrated that an early increase in body temperature within 24 hours after EVT was associated with sICH and worse long term outcomes [34]. Neutrophil-lymphocyte ratio may be yet another novel predictor of outcome after EVT that requires further study [35]. Other imaging features, such as percent insular ribbon infarction, may also play a role in understanding infarct growth and long term outcomes [36].

While there have been some prior analyses of the relationship between collaterals and 90-day outcomes, the data are mixed highlighting the need for further research [18, 32, 37]. One recent study utilized a more complicated cerebral collateral cascade (CCC), measuring the arterial, tissue, and venous phases [38]. CCC+ (good collaterals in all 3 phases) had median 90-day mRS 1, CCC- (poor for all phases) had median mRS 5, and other had median mRS 4. Our scoring system yielded remarkably similar findings: symmetric had median mRS 1, malignant had median mRS 4.5, and other had median mRS 3. However, our system does not require CT perfusion or any complicated data processing, and it has significant advantages compared to CT perfusion [16]. The advantages include: the eradication of the extra time needed to collect, process and interpret the perfusion data; elimination of the need for extra contrast administration; avoidance of the high additional radiation dose; and minimizing technical issues such as timing of the imaging with respect to contrast injection.

The mechanism of the relationship between collaterals and clinical outcomes may be explained by presentation infarct volume and infarct growth. Indeed, infarct volume is one of the strongest determinants of 90-day outcomes, even among patients who undergo EVT [39–41]. We have previously shown that patients with low infarct growth rates (slow progressors) were suitable for relatively late EVT [9, 16]. Furthermore, worse collaterals were an independent determinant of both greater presentation infarct volume and infarct growth rate, even when controlling for other variables including occlusion site, NIHSS, and age [16]. Beyond presentation, continued infarct growth may be important for outcomes even among those who achieve successful reperfusion with EVT [42, 43]. We previously demonstrated that collateral patterns continue to affect infarct growth up to 48 hours in patients not treated with EVT, suggesting there is no “collapse” of collaterals over this time [44].

There are several limitations of our study to consider. First, patients were identified, and collateral patterns were evaluated retrospectively. However, bias was minimized by including all EVT patients with studies that met the inclusion criteria over the 2-year study period; and the neuroradiologists that graded the collateral patterns were blinded to clinical history and clinical outcomes. There may be some selection bias for patients treated with EVT. Furthermore, since CTP or MRI were not used in the early 6-hour window and variably used in the extended 6–24-hour window in this cohort of patients, it was not possible to compare them to collateral patterns. Additionally, collaterals were assessed on the arterial phase images only, consistent with previously described methodology. This was by design since the CTA acquisition protocol and interpretation is straightforward and easy to implement at primary referral centers. However, our single stroke network design may limit generalizability. Also, the “other” collateral pattern category was heterogenous, with some patients rated as closer to symmetric and others rated as closer to malignant. Despite these potential limitations, this simple three-category classification system is easy to learn, easy to use, robust, and immediately translatable.

Importantly, there are several implications that derive from this study. Our classification approach for collaterals has been previously described [16], and is consistent with other studies using a three-category approach [45]. While CTP can be used to estimate collaterals [17, 45], CTA is more widely available, has no threshold dependence, and allows direct visualization of the cerebral vessels. The use of CTA only also eliminates the need for an additional contrast dose, avoids a substantially higher radiation dose, minimizes timing issues related to scanning after contrast injection, and is associated with reduced cost. Our simple classification system is robust and immediately translatable. There is growing data that extended window EVT is beneficial even without advanced imaging [46]. A simple non-contrast CT and single arterial phase CTA may be sufficient to triage most patients for EVT. Understanding collaterals may also aid in decision making for the inter-hospital transfer of patients to EVT-capable centers [47–49]. Moreover, in many smaller hospitals, MRI or CTP are not readily available [12, 50].

In conclusion, a symmetric collateral pattern is a robust predictor of a 90-day favorable outcomes after EVT in a real-world cohort of patients with LVO stroke. Further prospective studies are needed to confirm the value of collateral patterns in EVT transfer decisions and prognostication.
